# Supplementary material for: Multilocus Genetic Profile Reflecting Low Dopaminergic Signaling Is Directly Associated with Obesity and Cardiometabolic Disorders Due to Antipsychotic Treatment
Source: Pharmaceutics. 2023 Aug 14;15(8):2134. doi: 10.3390/pharmaceutics15082134 (PMC10459305; doi:10.3390/pharmaceutics15082134)
Supplement: Supplementary file 1 [file pharmaceutics-15-02134-s001.zip › pharmaceutics-2505574-supplementary.pdf]

pharmaceutics

MDPI

Supplementary Materials: Multilocus Genetic Profile Reflecting Low Dopaminergic Signaling Is Directly Associated with Obesity and Cardiometabolic Disorders Due to Antipsychotic Treatment

Aurora Arrue, Olga Olivas, Leire Erkoreka, Francisco J. Alvarez, Ainara Arnaiz, Noemí Varela, Ainhoa Bilbao, J. J. Rodríguez, M Teresa Moreno-Calle, Estibaliz Gordo, Elena Marín, Javier García-Cano, Estela Saez, Miguel A. Gonzalez-Torres, Mercedes Zumárraga and Nieves Basterreche

Table S1. Description of primers, probes, and PCR conditions used for identifying polymorphisms in the studied genes.

SNPs	PCR Components	C	V final (µl)	Real-time PCR conditions
*DRD2*rs1799732<br>*DRD2*rs6277<br>*COMT*rs4680	Primers and probes: Commercial kit TaqMan SNP Genotyping Assays, (Thermo Fisher Scientific, Waltham, MA, USA)	20x	0.25	95°C 2 mins<br>95°C 5 sec and<br>60°C 10 secfor<br>36 cycles
	Taq polymerase Sso Advanced (BioRad, Hercules, CA, USA)	1x	2.5	
	Water		2.25	
	DNA template		0.5	
DAT1-VNTR (40pb)	Primers: F:5′-AGGTCAGGCTACTGCCACTC-3′	3 µM	1.5	95°C 2 mins<br>95ºC 20 sec,<br>62°C 10 sec and<br>72°C 10 sec for<br>30 cycles
	R:5′-CCGGCAGAGTGTTCGTCTG-3′	3 µM	1.5	
	dNTPs: 4 X 10 mM	4 x 0.2 mM	0.5	
	Taq polymerase Kapa 2G Robust	1 unit	0.2	
	CG buffer (Kapa Biosystems, Wilmington, MA, USA)	1X	5	
	Water		15.3	
	DNA template		1	
DRD4-VNTR (48 pb)	Primers: F: 5′-GCGACTACGTGGTCTACTCG-3′	3 µM	1.5	95°C 3 minutes<br>95ºC 20 sec,<br>62°C 10 sec and<br>72°C 10 sec for<br>30 cycles
	R: 5′-AGGACCCTCATG GCCTTG-3′	3 µM	1.5	
	dNTPs: 4 X 10 mM	4 x 0.2 mM	0.5	
	Taq polymerase Kapa 2G Robust	1 unit	0.2	
	CG template (Kapa Biosystems, Wilmington, MA, USA)	1X	5	
	Water		15.3	
	DNA template		1	

SNP: single-nucleotide polymorphism, DRD2: dopamine receptor type 2, COMT: Catechol O-methyltransferase, VNTR: variable number of tandem repeats polymorphism, DAT1: DA transporter, DRD4: Dopamine receptor type 4, C: Concentration, V: Volume.

Table S2. Position and genotypes frequency of the selected polymorphisms.

Gen	Polymorphism	C[1]	Region	Position	Genotype	n	%
DRD2	rs1799732/ -141C InsDel[2]	11	5′	NG_008841.1:g.4750dup **-**>C	InsIns	243	85.3
					InsDel	42	14.7
					DelDel	0	
	rs6277	11	Exon 7	NG_008841.1:g.67543 **C**>T	TT	96	33.7
					CT	144	50.5
					CC	45	15.8
	rs4680/ Val158Met	22	Exon 3	NG_011526.1:g.27009G>**A**	GG	79	27.7
					AG	147	51.6
					AA	59	20.7
DAT1[3] (***SLC6A3***)	*DAT1*-VNTR	5	3´-UTR	NC_000005.10(1392794-1445440)	9R9R	36	12.6
					9R10R	124	43.5
					10R10R	125	43.9
DRD4[4]	*DRD4*-VNTR	11	Exon 3	NC_000011.10(637269-640706)	DRD4-S[5]	176	61.8
					DRD4-L[6]	109	38.2

[1] C: Chromosome. [2] No individual was a carrier of the DelDel genotype of DRD2rs179932 (-141C InsDel). In bold, the allele with the lowest frequency in the Iberian population. [3] 40 base pairs can be present in 3 to 11 repeats (3R-11R), being the 9 or 10 repeats the most common in European population. [4] 48 base pairs can be presents in 2 to 11 repeats (2R-11R), being the 2R, 4R and 7R alleles the most common in European population. [5] DRD4-S: genotypes formed by two alleles < 7R. [6] DRD4-L: genotypes formed by one or two alleles ≥ 7R.

Table S3. Statistical power χ^2 and mean comparisons.

		BMI	W	SBP	DBP	TG	HDL	GLU
DRD2	χ^2	8.122	1.199	0.440	1.044	1.254	1.052	0.898
**rs1799732**	n	284	283	283	283	283	283	282
	df	1	1	1	1	1	1	1
	df*	1	1	1	1	1	1	1
	w	0.1691	0.0651	0.0394	0.0607	0.0666	0.0610	0.0564
	Power	0.8132	0.1946	0.1018	0.1755	0.2014	0.1765	0.1575
**rs6277**	χ^2	0.208	0.339	2.433	4.207	0.856	1.878	1.242
	n	284	283	283	283	283	283	282
	df	2	2	2	2	2	2	2
	df*	1	1	1	1	1	1	1
	w	0.0271	0.0346	0.0927	0.1219	0.0550	0.0815	0.0664
	Power	0.0660	0.0764	0.2671	0.4342	0.1201	0.2139	0.1545
COMT	χ^2	1.390	0.976	1.911	2.682	5.697	3.555	4.318
**rs4680**	n	284	283	283	283	283	283	282
	df	2	2	2	2	2	2	2
	df*	1	1	1	1	1	1	1
	w	0.0670	0.0587	0.0829	0.0973	0.1419	0.1121	0.1237
	Power	0.1682	0.1306	0.2171	0.2909	0.5606	0.3742	0.4443
DAT1-	χ^2	4.241	6.049	4.574	9.823	12.699	4.144	3.505
**VNTR**	n	284	283	283	283	283	283	282
	df	2	2	2	2	2	2	2
	df*	1	1	1	1	1	1	1
	w	0.1222	0.1462	0.1271	0.1863	0.2119	0.1209	0.1115
	Power	0.4373	0.5878	0.4669	0.8081	0.9012	0.4286	0.3694
DRD4-	χ^2	3.539	3.765	0.209	0.001	0.842	0	1.043
**VNTR**	n	284	283	283	283	283	283	282
	df	1	1	1	1	1	1	1
	df*	1	1	1	1	1	1	1
	w	0.1116	0.1153	0.0272	0.0017	0.0545	0	0.0608
	Power	0.4686	0.4923	0.0742	0.0501	0.1506	0.0501	0.1754
**MLGP**	Mean 1 (SD 1)	2.53 (0.91)	2.46 (0.89)	2.29 (0.87)	2.42(0.89)	2.42 (0.87)	2.36 (0.90)	2.32 (0.86)
	Mean 2 (SD 2)	2.21 (0.84)	2.18 (0.87)	2.32 (0.91)	2.13 (0.84)	2.13 (0.86)	2.25 (0.86)	2.27 (0.92)
	n 1*/n 2*	68/173	105/136	151/89	140/100	139/102	111/129	171/69
	d	0.3632	0.2847	0.0365	0.3399	0.3352	0.1317	0.0555
	Power	0.7147	0.5883	0.0585	0.7341	0.7261	0.1733	0.0673

* 15% lower since the test used is actually the Mann-Whitney U test. $w = \sqrt{\frac{\chi^2}{n \cdot df^*}}$;

$df^* = \min(c-1, r.1)$; $d = \frac{\bar{X}_1 - \bar{X}_2}{\sqrt{\frac{SD_1^2 + SD_1^2}{2}}}$

Table S4. Statistical power regression models.

**Dependent variable**	**Regression logistic model**	**p-value**	**Predictor variable**	**N**	**Nag R^2**	**Power**
**BMI**	***DRD2*rs1799732 + illness duration**	0.05	2	266	0.0837	0.9955
	***DAT1*-VNTR + illness duration**	0.05	2	266	0.0667	0.9802
	**MLGP + illness duration**	0.05	2	266	0.0759	0.9909
**W**	***DAT1*-VNTR + sex**	0.05	2	283	0.1008	0.9995
	***DRD4*-VNTR +sex**	0.05	2	283	0.0954	0.9991
	**MLGP + sex**	0.05	2	283	0.0994	0.9994
**DBP**	***DAT1*-VNTR + age**	0.05	2	265	0.0752	0.9901
	**MLGP + age**	0.05	2	282	0.0660	0.9845
**TG**	***DAT1*-VNTR + onset**	0.05	2	265	0.0989	0.9989
	**MLGP + onset**	0.05	2	265	0.0632	0.9732

BMI: Body Mass Index, DRD2: dopamine receptor type 2, DAT1: DA transporter, VNTR: variable number of tandem repeats polymorphism, MLGP: Multilocus Genetic Profile Score, W: waist circumference, DRD4: Dopamine receptor type 4, DBP: Diastolic blood pressure, TG: Triglycerides

Table S5. Clinical-demographic distribution of patients according to the presence of obesity, hypertension, dyslipemia and hyperglycemia. Association analysis.

		**BMI** (n=284)		**W** (n=284)		**SBP** (n=283)		**DBP** (n=283)		**TG** (n=283)		**HDL** (n=283)		**Glu** (n=282)	
		n	%	n	%	n	%	n	%	n	%	n	%	n	%
		204	71.8	160	56,5	105	37.1	118	41.4	120	42.4	152	53.7	81	28.7
**Sex** (n=285)	Woman (n=97)	72	75.0	69	71.9	32	33.3	43	44.8	37	38.9	55	57.9	35	36.5
	Men (n=188)	132	70.2	91	48.7	73	39.5	75	40.1	83	43.6	97	51.6	46	24.9
	χ²*, *p	*0.72*	*0.396*	*13.9*	***<0.001***	*0.66*	*0.418*	*0.40*	*0.529*	*0.70*	*0.478*	*0.77*	*0.380*	*3.70*	***0.054***
**Tobacco** (n=284)	No (n=94)	70	75.3	58	62.4	37	39.4	38	40.4	33	35.5	44	47.3	33	35.1
	Yes (n=190)	134	70.5	102	54.0	67	35.6	79	42.0	86	45.5	108	57.1	48	25.7
	χ²*, *p	*0.48*	*0.487*	*1.47*	*0.226*	*0.23*	*0.631*	*0.02*	*0.898*	*2.18*	***0.141***	*2.04*	*0.153*	*2.27*	*0.131*
**Alcohol** (n=283)	No (n=238)	173	73.3	137	58.3	88	37.6	102	43.4	100	42.3	124	52.8	68	28.9
	Occasional (n=45)	30	66.7	23	51.1	16	35.6	15	33.3	18	40.0	27	60.0	13	28.9
	χ²*, *p	*0.75*	*0.386*	*0.74*	*0.486*	*0.00*	*0.958*	*1.14*	*0.286*	*0.02*	*0.896*	*0.57*	*0.449*	*0.00*	*1.00*
**Cannabis** (n=284)	No (n=244)	175	72.0	140	57.9	91	37.6	104	43.0	102	42.0	128	53.1	70	31.8
	Occasional (n=40)	29	72.5	20	50.0	13	32.5	13	32.5	17	43.6	24	60.0	11	10.3
	χ²*, *p	*0.00*	*1.00*	*0.57*	*0.450*	*0.20*	*0.658*	*1.15*	*0.784*	*0.00*	*0.988*	*0.74*	*0.391*	*0.00*	*1.00*
**AP treatment** (n=284)	Monotherapy (n=169)	118	69.8	91	54.4	54	32.3	64	38.3	65	38.9	84	50.3	53	31.7
	Polytherapy (n=115)	85	74.1	68	59.6	51	44.3	54	48.1	54	47.0	68	59.1	52	45.2
	χ²*, *p	*0.54*	*0.463*	*0.62*	*0.430*	*3.71*	***0.054***	*1.75*	*0.186*	*1.48*	*0.223*	*1.80*	*0.180*	*9.24*	***0.002***
**Con. Treat.** (n=282)	No (n=78)	61	77.2	44	57.1	31	40.8	31	40.8	29	37.2	36	46.2	22	28.6
	Yes (n=205)	143	70.4	115	56.4	73	35.6	87	42.4	90	44.3	116	57.1	59	29.1
	χ²*, *p	*0.99*	*0.321*	*0.01*	*1.00*	*0.43*	*0.509*	*0.01*	*0.910*	*0.91*	*0.341*	*2.32*	***0.128***	*0.00*	*1.00*

…/…

Table S5 cont... Clinical-demographic distribution of patients according to the presence of obesity, hypertension, dyslipemia and hyperglycemia. Association analysis.

		**BMI** (n=284)		**W** (n=283)		**SBP** (n=283)		**DBP** (n=283)		**TG** (n=283)		**HDL** (n=283)		**Glu** (n=282)	
		n	mean±sd	n	mean±sd	n	mean±sd	n	mean±sd	n	mean±sd	n	mean±sd	n	mean±sd
**Age , y**	normal P.	80	45.0±12.0	123	42.6±11.2	177	42.4±10.0	164	42.4±10.6	163	43.1±11.0	131	43.7±11.6	201	42.6±10.6
	altered P.	203	43.3±10.2	160	44.7±10.3	105	46.8±10.6	118	45.7±10.7	119	44.8±10.3	151	43.9±9.9	81	47.1±10.3
	Stu.-t test		0.263		**0.101**		**0.002**		**0.012**		0.200		0.894		**0.001**
**Onset, y**	normal P.	74	21.1±6.6	116	22.0±8.0	166	22.6±7.7	157	23. 1±8.2	152	22.0±7.7	125	22.4±8.3	187	23.1±8.0
	altered P.	192	23.8±8.5	149	23.8±8.1	99	23.6±8.7	108	22.9±11.2	113	24.5±8.5	140	23.7±8.0	77	23.0±8.0
	M-W *U*		**0.066**		**0.086**		0.326		0.932		**0.028**		**0.126**		0.916
**Illness duration, y**	normal P.	74	23.7±11.5	116	20.5±10.9	166	19.5±10.3	157	19.3±10.7	152	21.0±11.1	125	21.1±11.5	187	19.4±11.0
	altered P.	192	19.5±.10.7	149	21.0±11.2	99	22.9±12.0	108	22.8±11.2	113	20.4±11.1	140	20.2±10.7	77	24.2±10.6
	M-W *U*		**0.008**		0.769		**0.038**		**0.013**		0.765		0.615		**0.001**
**AP daily doses, mg**	normal P.	79	757.8±645.9	121	698.5±559.6	174	665.2±498.5	101	651.0±492.0	161	668.8±511.7	127	633.4±379.6	197	657.3±474.1
	altered P.	201	634.8±333.2	158	649.8±336.5	105	685.1±343.3	118	702.3±374.0	118	671.1±342.3	152	700.0±496.0	81	706.1±375.8
	M-W *U*		0.374		0.871		0.165		**0.070**		0.293		0.189		0.152
**AP Treatment time, m**	normal P.	80	27.1±41.3	123	26.0±41.1	177	25.3±40.2	164	24.3±38.2	163	23.9±37.1	130	25.3±39.2	200	25.3±38.0
	altered P.	203	28.2±41.7	159	30.0±42.0	105	31.8±43.0	118	32.5±45.2	119	32.3±44.9	152	29.3±42.0	81	33.3±47.1
	M-W *U*		0.524		**0.124**		**0.013**		**0.047**		**0.056**		0.306		0.232

Data are presented as n: number of patients with altered values and %. BMI: Body Mass Index, W: Waist circumference, SBP: Systolic blood pressure, DBP: Diastolic blood pressure, TG: Triglycerides, HDL: High Density lipoproteins, Glu: Glucose, AP: antipsychotic, Con. treatment: concomitant treatment (euthymizers, antidepressants, anxiolytics), y: years, m: months, normal P: normal values parameter, altered P: altered values parameter, Stu.-t test: Student-t test, M-W *U*: Mann Whitney–U test, n=total number of patients with altered values for each parameter.

Table S6. Association analysis. Frequency distribution of the genotypes of the studied polymorphisms according to presence/absence of altered BMI, waist circumference, blood pressure and TG, HDL or glucose levels using WHO[1] or ATP-III[2] criteria in antipsychotic treatment patients.

				**BMI[1]**		**W[2]**		**SBP[2]**		**DBP[2]**		**TG[2]**		**HDL[2]**		**Glu[2]**	
				No 178	A 105	No 178	A 105	No 178	A 105	No 178	A 105	No 178	A 105	No 161	A 152	No 201	A 81
**Gene**	**SNP**	**Genotype**	n	n	n	n	n	n	n	n	n	n	n	n	n	n	n
DRD2	rs1799732	InsIns	243	60	182	101	140	156	87	137	104	135	104	108	133	168	72
		InsDel	42	20	22	22	20	24	18	28	14	28	14	23	19	33	9
		X^2, p		***8.12***	***0.004***	*1.20*	*0.274*	*0.44*	*0.507*	*1.04*	*0.307*	*1.25*	*0.263*	*1.05*	*0.305*	*0.90*	*0.343*
	rs6277	TT	96	26	70	39	56	60	36	48	48	51	43	48	46	67	26
		CT	144	42	101	64	79	94	48	88	54	84	60	61	83	105	39
		CC	45	12	33	20	25	24	21	29	16	28	17	22	23	29	16
		X^2, p		*0.21*	*0.901*	*0.34*	*0.844*	*2.43*	*0.296*	*4.21*	*0.122*	*0.86*	*0.650*	*1.88*	*0.391*	*1.24*	*0.537*
COMT	rs4680	MetMet	59	13	46	25	34	32	26	35	23	30	29	26	33	37	22
		ValMet	149	44	103	60	85	94	52	90	56	94	52	75	71	104	43
		ValVal	79	23	55	38	41	52	27	40	39	39	39	30	48	60	16
		X^2, p		*1.39*	*0.449*	*0.98*	*0.614*	*1.91*	*0.385*	*2.68*	*0.262*	*5.70*	*0.058*	*3.55*	*0.169*	*4.32*	*0.115*
DAT1	VNTR	9R9R	36	5	30	9	27	17	19	13	23	11	25	11	25	21	15
		9R10R	124	35	89	55	69	82	41	71	52	74	50	61	63	92	32
		10R10R	125	40	85	59	64	79	45	81	43	78	45	59	64	88	34
		X^2, p		*4.24*	*0.120*	***6.05***	***0.049***	*4.58*	*0.102*	***9.82***	***0.007***	***12.7***	***0.002***	*4.14*	*0.126*	*3.50*	*0.173*
**DRD4-S**		2 allele	176	52	119	84	90	113	63	102	74	105	70	81	94	129	46
**DRD4-L**		1,2 allele	109	23	85	39	70	65	42	63	44	58	50	50	58	72	35
		X^2, p		*3.54*	*0.060*	3.76	0.052	*0.21*	*0.648*	*0.001*	*0.977*	*1.08*	*0.298*	*0.00*	*0.999*	*1.04*	*0.307*

BMI: Body Mass Index, W: Waist circumference, SBP: Systolic blood pressure, HDL: High Density lipoproteins, Glu: Glucose, SNP: Single-nucleotide polymorphism, VNTR: Variable number of tandem repeats polymorphism, DRD2: Dopamine receptor type 2, COMT: Catechol O-methyltransferase, DAT1: Dopamine transporter, DRD4: Dopamine receptor type 4, n: Number of patients in each group, No: Normal values, A: Altered values.
